# Probiotics and their Metabolites Reduce Oxidative Stress in Middle-Aged Mice

**DOI:** 10.1007/s00284-022-02783-y

**Published:** 2022-02-14

**Authors:** Wen-Yang Lin, Jia-Hung Lin, Yi-Wei Kuo, Pei-Fang Rose Chiang, Hsieh-Hsun Ho

**Affiliations:** 1 Department of Research and Design, Bioflag Biotech Co., Ltd., 4F.C2, No.17, Guoji Rd, Xinshi Dist, Tainan City, 744 Taiwan; 2 Department of Psychology, Jacobs University Bremen, Campus Ring 1, Vegesack, 28759 Bremen, Germany

## Abstract

Aging is an irreversible physiological degradation of living organisms. Accumulated oxidative stress and dysbiosis accelerate aging. Probiotics such as *Lactobacillus* and *Bifidobacterium* and their fermented metabolites (postbiotics) have been discovered to exhibit antioxidative activities that regulate oxidative stress and protect cells from oxidative damage. We screened selected *Lactobacillus* and *Bifidobacterium* strains and their postbiotics for potential antioxidative activity by using DPPH (2,2-Diphenyl-1-picrylhydrazyl) assay. Strains with their metabolites were selected for mixed formula in experiments involving aging mice. The aged groups presented higher oxidative stress in the brain, liver, heart, and kidney than did young mice. However, treatment with probiotic strains and their postbiotics elevated antioxidative levels, especially in the high-dose probiotics plus postbiotics group. Next-generation sequencing data revealed positive microbiota alterations of *Lactobacillus* and *Bifidobacterium* and *Akkermansia* in the gut. *Lactobacillus johnsonii* and *Akkermansia muciniphila* exhibited effective enlargement of relative abundance. Besides, high-dose probiotics and high-dose probiotics plus postbiotics showed significant elevation in serum SCFAs, especially in butyrate. In conclusion, the formula containing *Bifidobacterium animalis* subsp. *infantis* BLI-02, *Bifidobacterium breve* Bv889, *Bifidobacterium bifidum* VDD088, *B. animalis* subsp. *lactis* CP-9, and *Lactobacillus plantarum* PL-02 and their metabolites may benefit aged people’s health.

**Supplementary Information:**

The online version contains supplementary material available at 10.1007/s00284-022-02783-y.

## Introduction

Aging is a general, progressive, cumulative, and harmful physiological decline. Because of differences between individuals, aging cannot be described with a single or simple model [1]. However, as research progresses, researchers have discovered that human aging is closely related to reactive oxygen species in the body [2]. Oxidative free radicals can cause damage to DNA, proteins, lipids, and other molecules of cells, causing the gradual loss of physiological functions and diseases including cardiovascular disease and cancer [3]. Numerous studies have demonstrated that oxidative free radicals are a main factor in aging in internal organs [4]. Therefore, research on new antioxidant substances that delay aging is the principal focus of antiaging research.

Over 10^14^ microorganisms inhabit the human gastrointestinal (GI) tract. The number of GI bacterial cells is 10 times that of human cells, with microbial genomic content numbering over 3 million, whereas the human genome contains approximately 23,000 genes [5]. Scientists have discovered microbiota changes in older people [6], whose number of beneficial gut bacteria, including *Lactobacilli* and *Bifidobacteria*, are greatly reduced and certain facultative anaerobes and gram-negative bacteria (mainly *Enterobacteria*) multiply. The shift of gut microbiota may lead to small bowel bacterial overgrowth followed by symptoms such as diarrhea, nutrient malabsorption, and weight loss in aged people [7].

Accumulated findings have demonstrated that probiotic strains of *Lactobacilli* and *Bifidobacteria* possess antioxidant properties [8]. The metabolites secreted by probiotic strains (postbiotics) may play key roles in promoting antioxidative activity. An animal study confirmed that probiotic metabolites improve serum antioxidant activity and upregulate hepatic antioxidant enzymes [9]. Moreover, a double-blind clinical trial revealed that probiotic supplementation in patients with Alzheimer disease improved cognitive function [10]. Moreover, the food safety of lactic acid bacteria has been validated in clinical trials [11]. However, unpredicted side effects have been reported in some effective antioxidants, such as resveratrol. For example, in clinical trials, taking high doses of resveratrol (2.5–5.0 g/day) led to symptoms such as nausea, flatulence, abdominal discomfort, and diarrhea [12]. Anticoagulant effects were also revealed, and it may interfere with the metabolism of drugs in the liver [13].

Probiotic strains together with their fermented products can be considered candidate nutritional supplements for downregulating the oxidative stress that induces the aging process. DPPH (2,2-diphenyl-1-picrylhydrazyl) is an oxidative free radical, which has been widely used to select antioxidative substances or organisms before animal study [14, 15]. In this study, we screened effective antiaging probiotic strains through in vitro antioxidative assay (DPPH assay). We then used an animal model to validate the antioxidative properties of the probiotic strains with their metabolites. Finally, the microbiota shifts caused by taking the probiotic products were detected and confirmed using the next generation sequencing technique (NGS).

## Materials and Methods

### Probiotic Strains and Culturing of Strains

All the probiotic strains were sourced from human intestinal tract and obtained from Bioflag Biotech Co. (Tainan, Taiwan). The strains were preserved in the China Center for Type Culture Collection (CCTCC) and the China General Microbiological Culture Collection Center (CGMCC). The deposit numbers for *Bifidobacterium animalis* subsp. *infantis* BLI-02, *Bifidobacterium breve Bv889*, *Bifidobacterium bifidum* VDD088, *B. animalis* subsp. *lactis* CP-9, and *Lactobacillus plantarum* PL-02 are CGMCC-15212, CGMCC-16145, CGMCC-15211, CCTCC-M2014588, and CGMCC-20485, respectively. *Lactobacillus lactis* L-87, *Lactobacillus rhamnosus* L-13, *Enterococcus faecium* L-38, *Lactobacillus gasseri* L-2, *Lactobacillus paracasei* L-134, *L. paracasei* L-30 and *Streptococcus thermophilus* L-243 were obtained from Bioflag Biotech Co. (Tainan, Taiwan).

The probiotic lactic acid bacteria strains were stored at − 80 °C with 20% glycerol. MRS broth (Difco, BD™, New Jersey, United States) containing 0.05% cysteine was used to activate the strains at 37 °C (24 h) twice. The postbiotic products were gained from fermentation of the strains. A liquid medium of 5%–30% milk and 1%–10% soybean meal was used for fermenting the probiotics. The fermentation broth was finally purified through centrifugation, filtration, and heat sterilization and dried into powder. The powder of the fermentation broth was stored at room temperature.

### Screening Potential Probiotic Strains Through Antioxidative Assay

DPPH (2,2-Diphenyl-1-picrylhydrazyl) is a stable free radical molecule whose highest absorption value is at a wavelength of 517 nm in a methanol solution. When DPPH free radicals interact with antioxidants, the antioxidants provide hydrogen protons to scavenge free radicals, and DPPH free radicals lose their blue–violet characteristics and their light absorption decreases. The decrease in OD_517_ value was used to determine the free radical scavenging ability of the tested lactic acid bacteria strains.

The method for detecting the free radical scavenging ability of the lactic acid bacteria strains or their metabolites was as follows. Common lactic acid bacteria strains (approximately 2 × 10^9^ colony-forming units [CFU], optical density [OD] approximately 2) or a strain’s own fermentation broth were mixed 1:1 with 0.2 mM DPPH in methanol. After being mixed, the solution was maintained at room temperature in the dark for 30 min for reaction. After centrifuging (12,000 rpm, 2 min) at 4 °C and transferring 200 μL of supernatant to a 96-well plate, the OD_517_ value was measured. The *Streptococcus* thermophiles SY-66 strain without antioxidant activity was used as a negative control (approximately 2 × 10^9^ CFU, OD approximately 2) and diluted water as a blank control. The calculation formula used for free radical scavenging ability is as follows:

$${\text{Free radical scavenging capacity}} = {\text{OD}}_{{{\text{blank}}}} \, - \,{\text{OD}}_{{{\text{sample}}}} /{\text{ OD}}_{{{\text{blank}}}} \times {1}00\%$$

where OD_sample_ is the absorbance value of the tested sample and OD_blank_ is the absorbance value of the blank group. Vitamin C (10 µg/mL) was used as a positive control.

### Mice and Ethics Statement

Animal experiments and protocols were in compliance with the National Institute of Health’s *Guide for the Care and Use of Laboratory Animals*. The protocols were approved (the Approval Number—IACUC no.NLAC(TN)-108-D-002) by animal ethics committee of National Laboratory Animal Center (Taipei, Taiwan). C57BL/6 mice (age: 2 months) were used for the study. The animals were housed in groups of six in sterilized cages fitted with filter cage tops and fed with sterilized food and water. The housing environment was strictly monitored and maintained under 22 ± 2 ℃ and 62% ± 5% humidity. Each group was entrusted to the National Laboratory Animal Center (Taipei, Taiwan) during the experiment, and tube feeding was administered daily according to the experimental design.

### Animal Experimental Design

The experimental mice naturally developed and were divided into nine groups (each group with four mice, two male plus two female) as follows: 2-month-old (2 M), 10-month-old (10 M), 13-month-old (13 M), and 16-month-old (16 M) groups; among the 10-month-old groups were a low-dose probiotics group (1.03 × 10^9^ CFU/kg, per mouse, daily dose), a low-dose probiotics and postbiotics group (1.03 × 10^9^ CFU/kg plus 20.5 mg/kg of postbiotics, per mouse, daily dose), a high-dose probiotics group (4.1 × 10^9^ CFU/kg, each mouse, daily dose), a high-dose probiotics and postbiotics group (4.1 × 10^9^ CFU/kg probiotics plus 20.5 mg/kg of postbiotics, per mouse, daily dose), and a positive control group fed general feed plus resveratrol (25 mg/kg mice/day). The 2 M, 10 M, 13 M, and 16 M groups were fed with general feed without extra treatment. The mice were sacrificed for the detection of oxidative and antioxidative elements in tissues.

The probiotic-treated groups and the positive control group were fed with treatments from the age of 10 months to 16 months. The dose of lactic acid bacteria ingested by the animals was estimated according to the initial experimental method published by the US Food and Drug Administration in 2005. The five probiotic strains were mixed at a ratio of 1:1:1:1:1 for daily feeding, and the postbiotics were mixed with the same five strains in the same ratio. After consecutive feedings for 6 months, the tissues of this “aging” group were also evaluated (Supplemental Fig. 1).

### Evaluating Oxidative and Antioxidative Level in Mice

All mice were sacrificed using CO_2_; next, the brain, heart, liver, and other tissues were removed with surgical instruments and placed in microcentrifuge tubes; 200 μL 0.4 M perchloric acid was added, and an ultrasonic homogenizer was used for homogenization (all procedures were performed on ice). Subsequently, the antioxidative levels in the mouse tissues were measured through superoxide dismutase (SOD) assay (Cayman Chemical Item No. 706002), glutathione peroxidase (GPx) assay (Cayman Chemical Item No. 703102), and catalase (CAT) assay (Cayman Chemical Item No. 707002). The oxidative levels were measured (and the protocol followed) through TBARS assay (Cayman Chemical Item No. 10009055), protein carbonyl colorimetric assay (Cayman Chemical Item No. 10005020), and New 8­OHdG Check ELISA (Item No. KOG-200SE, JalCA). Mitochondria DNA was extracted using a mitochondrial DNA isolation kit (Item No. K280-50, BioVision) for measuring 8-oxo-2'-deoxyguanosine (8­OHdG). All assays were conducted in accordance with the experimental standard procedures provided with the product. All oxidative and antioxidative indicators are consistent with the standard experimental test for antiaging health food identified by the Ministry of Health of the Republic of China (Taiwan).

### Evaluating Gut Microbiota Change Through the NGS Method

Mouse feces were collected after 6 months of consecutive treatment and immediately placed in a − 80 °C refrigerator. Subsequently, Quick-DNATM fungal/bacterial micropreparation reagent (ZYMO Research, USA) was used to extract the DNA from the stool samples. Diluent DNA in sterile water (5 ng/μL) was used for quality inspection through 1% agar gel electrophoresis. Next, we amplified the DNA fragments (16S rRNA, 16S V3-V4) by using polymerase chain reaction (PCR) through specific DNA-fragment targeting primers (Pusion High-Fidelity PCR Master Mix, New England Biolabs, USA). The purifying PCR generated DNA products by using AMPure XP beads (Beckman Coulter Genomics, USA). Then, an Illumina Nextera XT Index kit (Illumina, USA) was used to produce sample libraries, which were evaluated using a Qubit@2.0 Fluorometer (Thermo Scientific, USA) and sequenced on the Illumina MiSeq platform. The Greengenes database (http://greengenes.lbl.gov) was used for merging total reads, removing low-quality sequence, removing chimera sequence and clustering the OTU at 97% similarity. The CLC Microbial Genomics Module (Qiagen, Germany), basespace (illumine, USA) and Graphpad prism 8.1 (Graphpad Software, San Diego, CA, USA) were used to analyze all OTU sequences and diversity. The analysis procedure was according to previous study [16].

### Measurements of Serum Short-Chain Fatty Acids (SCFAs) Levels

Add 0.3 mL of deionized water to 0.03 g of feces samples for homogenization.

Centrifuge homogenized feces samples to isolate 150 ul of the supernatant. Add 50 ul of 50% sulfuric acid, 10 ul internal standard and 200 ul ether to feces samples and shake for 15 min. Centrifuge at 4 °C 9000 rpm for 10 min. The ether layer was added with anhydrous sodium sulfate (Na_2_SO_4_) for dehydration and then detected by GS/MS (Agilent 7890 gas chromatography mass spectrometer equipped with Agilent HP-FFAP capillary column, 30 m*250um*0.25um; Santa Clara, CA, United States). The GS/MS analysis conditions were as follows: injection volume 1 uL (Split 5:1), gas flow rate (1 mL/min), heating conditions (hold 80 °C for 1 min, then raised to 150 °C. Heating rate was 5 °C/min. After raising temperature to 230 °C, heating rate became 40 °C/min, hold on 12 min). Injection temperature was 240 °C. Transmission line temperature was 240 °C. Ion source temperature was 230 °C.The temperature of the quadrupole was 150 °C. The experimental protocol was followed previous study [17]. Serum SCFA level were presented as (%) = averaged SCFAs levels in treatment groups/averaged SCFAs levels in 16 M mice control.

### Statistics

The contents of each group were then analyzed (Graphpad Prism 8, Graphpad Software, San Diego, CA, USA).) using Brown-Forsythe ANOVA accompanied with Tamhane’s T2 (post hoc) to compare the differences between the groups. For the NGS analysis, heatmap figures were generated using Graphpad Prism 8.1. Statistical significance was indicated if *P < 0.05, **P < 0.01, ***P < 0.001, ****P < 0.0001*.*

## Results

### Screening Potential Probiotics Through In Vitro Antioxidative Testing

**Fig. 1:**
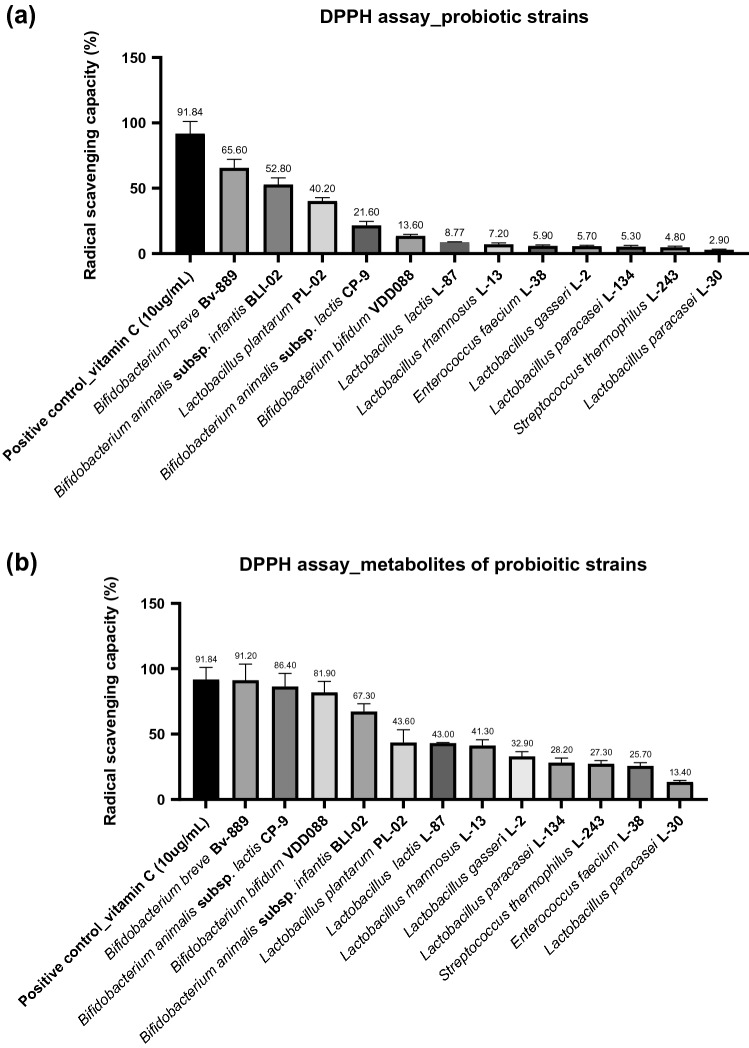
Screening potential antioxidative probiotics through in vitro radical scavenging capacity testing (DPPH assay). **a** antioxidative ability ranking of viable probiotic strains. **b** antioxidative ability ranking of metabolites of probiotic strains (postbiotics). The experimental probiotic strains were listed below: *B. animalis* subsp. *infantis* BLI-02, *B. breve* Bv889, *B. bifidum* VDD088, *B. animalis* subsp. *lactis* CP-9, *Lactobacillus plantarum* PL-02, *L. lactis* L-87, *L. rhamnosus* L-13, *E. faecium* L-38, *L. gasseri* L-2, *L. paracasei* L-134, *L. paracasei* L-30, and *S. thermophilus* L-243. 10 µg/ml of vitamin C was used as positive control

First, we used an in vitro DPPH assay to screen probiotic strains potentially possessing antioxidative ability. The five strains with superior antioxidative ability were Bv889 (65.6%), BLI-02 (52.8%), PL-02 (40.2%), CP-9 (21.6%), and VDD088 (13.6%). The antioxidative ability of the others was less than 10%. Vitamin C (10 µg/mL; 91.84%) was used as the positive control for the radical scavenging test (Fig. 1a). We further tested the antioxidative ability of the postbiotics. The postbiotics exhibited excellent antioxidative ability, among which that of Bv889 was 91.2%, CP-9 86.4%, Bf-668 81.9%, BLI-02 67.3%, and PL-02 43.6% (Fig. 1b). Subsequently, we selected the five trains with the highest antioxidative ability and their postbiotics (Bv889, CP-9, Bf-668, BLI-02, and PL-02) to treat aging mice.

### Confirming the Successful Setting of Naturally Aging Mouse Model

We used the murine model to investigate whether oxidative stress would be mitigated by probiotic treatment. Researchers have defined 2–6-month-old mice as equivalent to young human adults (~ 18–30 years), 10–16 months as human middle age (~ 38–49 years), and 18–24 months as human old age (~ 56–69 years) [18, 19]. We measured oxidative stress markers in mice from age 2 months to 16 months.

**Fig. 2:**
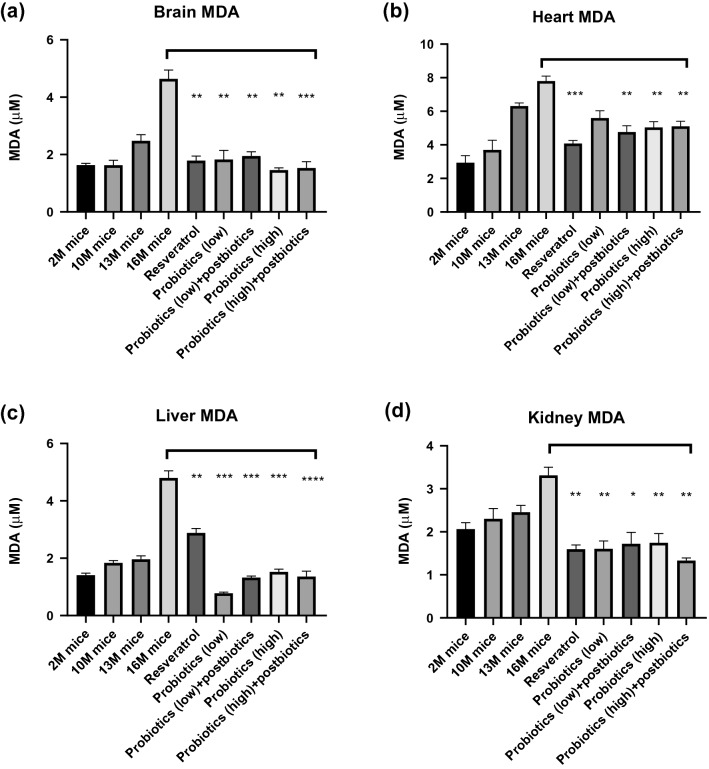
Probiotics reduced oxidative stress (lipid peroxidation level; MDA) in the **a** brain, **b** heart, **c** liver, and **d** kidney (probiotics administered for 6 months). Experimental mice were divided into nine groups. The groups 2 months old (2 M), 10 months old (10 M), 13 months old (13 M), and 16 months old (16 M) were not treated with probiotics. The 2 M mice were considered young mice and those older than 10 months were considered aged mice. Resveratrol was used as positive control for antiaging intervention. We began the probiotic treatment from the 10th month of age and treated the mice for 6 months. Treatment groups were compared with the 16 M group (vehicle control). *P < 0.05, **P < 0.01, ***P < 0.001

Because the experiment was conducted in a natural aging mouse model, observation of the change in oxidative stress indicators and antioxidant enzymes in the mice by age was necessary to confirm that the establishment of the natural aging model was successful. Therefore, prior to the start of the formal experiment, mice groups of 2 months, 10 months, 13 months, and 16 months of age were collected, and their brains, hearts, livers, and kidneys were tested for antioxidant enzymes, including SOD, CAT, and GPx, all of which gradually decline with age. We also examined a lipid, a protein, and a nucleic acid under peroxide attack by aging. The biological activity indicators included lipid oxide in propylene glycol (malondialdehyde, MDA), protein carbonyl after protein oxidation, and mitochondrial 8OHdG content, levels of which increase with age (Fig. 2; Supplemental Figs. 2-5).

### Brain, Heart, Liver, Kidney Antioxidative Levels Elevated in Middle-Aged Mice Taking Probiotics

According to the results of previous experiments, we fed different formulas of selected probiotic strains with their metabolites to 10-month-old mouse groups for 6 months (Fig. 2; Supplemental Figs. 2-5). Each group consisted of two male and female mice. The feeding groups were as described in Materials and Methods. The selected strains were mixed probiotics with strong antioxidant capacity according to prior in vitro experiments.

Oxidative stress markers MDA significantly decreased in brain, heart, liver and kidneyby taking probiotic formulas (Fig. 2a–d). Compared with the 16 months natural aging group (4.64 µM), brain MDA levels decreased in all the experimental groups, including the resveratrol control (1.78 µM, **P < 0.01), low-dose probiotics (1.82 µM, **P < 0.01), low-dose probiotics plus postbiotics (1.95 µM, **P < 0.01), high-dose probiotics (1.46 µM, **P < 0.01), and high-dose probiotics plus postbiotics (1.53 µM, ***P < 0.001) groups (Fig. 2a). Furthermore, high-dose treatment with probiotics and high-dose probiotics plus postbiotics significantly reduced protein C + and mitochondrial 8OHdG levels in the brain (Supplementary Fig. 2e).

Heart MDA levels decreased in all the experimental groups by comparing to 16 M middle-age group (7.79 µM), including the resveratrol control (4.08 µM, ***P < 0.001), low-dose probiotics (5.59 µM), low-dose probiotics plus postbiotics (4.76 µM, **P < 0.01), high-dose probiotics (5.03 µM, **P < 0.01), and high-dose probiotics plus postbiotics (5.11 µM, **P < 0.01) groups (Fig. 2b).

Liver MDA levels decreased in all the experimental groups by comparing to 16 M middle-age group (4.8 µM), including the resveratrol control (2.88 µM, **P < 0.01), low-dose probiotics (0.78 µM, ***P < 0.001), low-dose probiotics plus postbiotics (1.32 µM, ***P < 0.001), high-dose probiotics (1.52 µM, ***P < 0.001), and high-dose probiotics plus postbiotics (1.36 µM, ****P < 0.0001) groups (Fig. 2c). Finally, kidney MDA levels decreased in all the experimental groups by comparing to 16 M middle-age group (3.31 µM), including the resveratrol control (1.59 µM, **P < 0.001), low-dose probiotics (1.6 µM, **P < 0.01), low-dose probiotics plus postbiotics (1.72 µM, *P < 0.05), high-dose probiotics (1.74 µM, **P < 0.01), and high-dose probiotics plus postbiotics (1.33 µM, **P < 0.01) groups (Fig. 2d).

### Gut Microbiota Phylum Changes Among Middle-Aged Mice Fed Probiotics

We examined gut microbiota changes to verify whether the fed probiotics colonized in the gut and determine the influence on antioxidative levels of the colonizing probiotic strains. NGS was employed to analyze microbiota in mouse feces. We compared the microbiota of seven groups: 2 M (young age group without treatment), 16 M (middle-age group without treatment), the resveratrol treatment group (16 months old), the low-dose probiotics group (16 months old), the low-dose probiotics plus postbiotics group (16 months old), the high-dose probiotics group (16 months old), and the high-dose probiotics plus postbiotics group (16 months old).

**Fig. 3:**
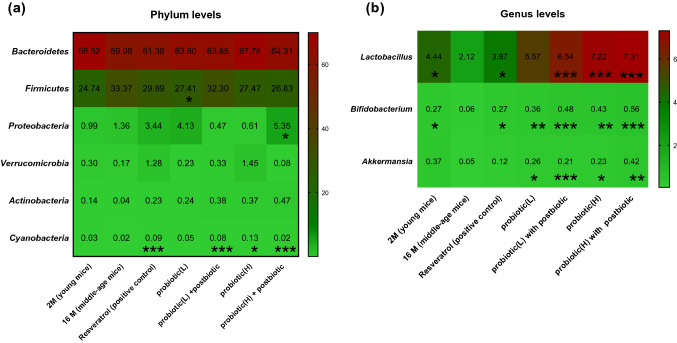
NGS analysis of gut microbiota change by taking probiotic product **a** in phylum level and **b** in genus level. The group 2 M was considered as young age mice. We start to feed probiotic treatment from 10th month and treatment last 6 months. Treatment groups were compared to the 16 M group (mice aged 16 months without any probiotic treatment, as vehicle control). Resveratrol was used as positive treatment control. *P* < 0.05*, *P* < 0.01**and *P* < 0.001*** stands for statistical difference

At the phylum level, the relative abundance of *proteobacteria* was significantly elevated in the high-dose probiotics plus postbiotics group (5.35%, *P* < 0.05) compared with the 16 M group (1.36%) (Fig. 3a). The relative abundance of *Actinobacteria* was 0.14% in 2 M and 0.043% in 16 M. However, treatment with resveratrol, low-dose probiotics plus postbiotics, high-dose of probiotics, and high-dose probiotics plus postbiotics significantly increased gut *Actinobacteria* to 0.22% (*P* < 0.001), 0.38% (*P* < 0.001), 0.37% (*P* < 0.05), and 0.46% (*P* < 0.001), respectively (Fig. 3a).

### Genus Abundance of *Lactobacillus*, *Bifidobacterium*, and *Akkermansia* Elevated by Probiotics

At the genus level, the relative abundance of *Lactobacillus* was 4.43% in 2 M, whereas it dropped significantly to 2.11% (*P* < 0.05) in the middle-age group with natural aging (Fig. 3b). However, after 6 months of intervention, the resveratrol, low-dose probiotics, low-dose probiotics plus postbiotics, high-dose probiotics, and high-dose probiotics plus postbiotics groups had elevated gut populations of *Lactobacillus* of 3.86% (*P* < 0.05), 5.56%, 6.54% (*p* < 0.001), 7.22% (*P* < 0.001), and 7.31% (*P* < 0.001), respectively (Fig. 3b). Similarly, the relative abundance of *Bifidobacterium* was 0.27% in the 2 M group but was significantly decreased to 0.05% (*P* < 0.05) in the middle-aged group. After 6 months of intervention, the resveratrol, low-dose probiotics, low-dose probiotics plus postbiotics, high-dose probiotics, and high-dose probiotics plus postbiotics groups exhibited elevated gut populations of *Lactobacillus* of 0.27% (*P* < 0.05), 0.35% (*P* < 0.01), 0.48% (*P* < 0.001), 0.42% (*P* < 0.01), and 0.56% (*P* < 0.001), respectively (Fig. 3b). Furthermore, the relative abundance of *Akkermansia* was 0.35% in 2 M, whereas it was significantly decreased to 0.04% (*P* < 0.05) in the middle-aged group. After 6 months of intervention, the resveratrol, low-dose probiotics, low-dose probiotics plus postbiotics, high-dose probiotics, and high-dose probiotics plus postbiotics groups contained elevated gut populations of *Lactobacillus* of 0.11%, 0.26% (*P* < 0.05), 0.2% (*P* < 0.001), 0.23% (*P* < 0.01), and 0.41% (*P* < 0.01), respectively (Fig. 3b).

### Probiotic Intervention Changed the Dispersion of Lactobacillus Species in Middle-Aged Mice

**Fig. 4:**
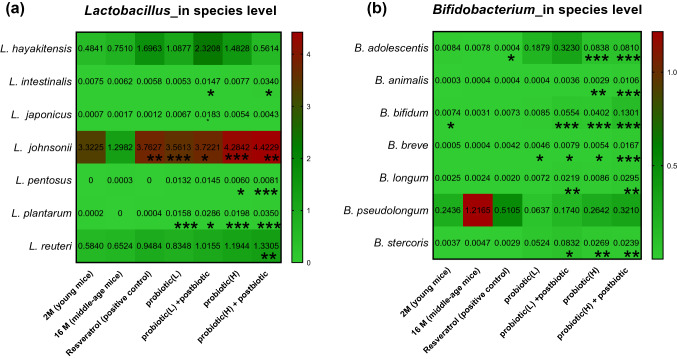
NGS analysis of gut microbiota change by taking probiotic product **a** in species of Lactobacillus and **b** in species of *Bifidobacterium*. The group 2 M was considered as young age mice. We start to feed probiotic treatment from 10th month and treatment last 6 months. Treatment groups were compared to the 16 M group (mice aged 16 months without any probiotic treatment, as vehicle control). Resveratrol was used as positive treatment control. *P* < 0.05*, *P* < 0.01**and *P* < 0.001*** stands for statistical difference

Next, The Greengenes database was used to classify the OTU of *Lactobacillus* and *Bifidobacterium* at 97% similarity. The abundance of *L. plantarum* was significantly expanded in the probiotic-treated groups, specifically, in the low-dose probiotics (0.01%, *P* < 0.001), low-dose probiotics plus postbiotics (0.02%, *P* < 0.05), high-dose probiotics (0.01%, *P* < 0.001), and high-dose probiotics plus postbiotics (0.03%, *P* < 0.001) groups (Fig. 4a). The abundance of *Lactobacillus intestinalis*, *Lactobacillus japonicas*, *Lactobacillus pentosus*, *Lactobacillus reuteri*, and *Lactobacillus johnsonii* was also significantly increased in the probiotic groups.

Among all species of *Lactobacillus* in the gut, *L. johnsonii* exhibited a dramatic change following probiotic treatment. The relative abundance of *L. johnsonii* was 3.32% in 2 M but decreased to 1.29% in the middle-aged group. After 6 months of intervention, the resveratrol, low-dose probiotics, low-dose probiotics plus postbiotics, high-dose probiotics, and high-dose probiotics plus postbiotics groups had elevated gut populations of *L. johnsonii*, of 3.76% (*P* < 0.01), 3.56% (*P* < 0.001), 3.72% (*P* < 0.05), 4.28% (*P* < 0.001), and 4.42% (*P* < 0.01), respectively (Fig. 4a).

### Probiotic Intervention Changed the Dispersion of Species of *Bifidobacterium* in Middle-Aged Mice

The abundance of each of *B. animalis*, *B. bifidum*, and *B. breve* in the gut was elevated by the mixed probiotic strains (Fig. 4b). The gut population of *B. animalis* significantly increased in the high-dose probiotics group (0.0029%, *P* < 0.01) and the high-dose probiotics plus postbiotics group (0.01%, *P* < 0.001). The abundance of gut *B. bifidum* was significantly elevated in the low-dose probiotics plus postbiotics (0.05%, *P* < 0.001), high-dose probiotics (0.04%, *P* < 0.001), and high-dose probiotics plus postbiotics (0.13%, *P* < 0.001) groups. Moreover, all probiotic treatment groups had greater abundance of *B. breve* in the gut; 0.004% (*P* < 0.05) in the low-dose probiotics, 0.007% (*P* < 0.05) in the low-dose probiotics plus postbiotics, 0.005% (*P* < 0.05) in the high-dose probiotics, and 0.016% in the high-dose probiotics plus postbiotics (*P* < 0.001) groups (Fig. 4b). Moreover, some gut *Bifidobacterium* populations different from the *Bifidobacterium* in the supplement of mixed strains also increased after treatment, including *Bifidobacterium adolescentis, Bifidobacterium longum*, and *Bifidobacterium stercoris.*

### High-Dose Probiotic Intervention Changed the Serum Short-Chain Fatty Acids (SCFAs) Distribution in Middle-Aged Mice

**Fig. 5:**
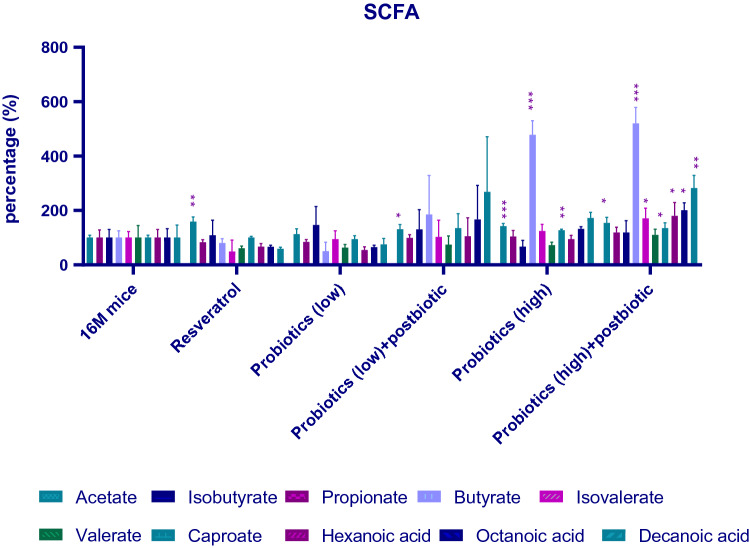
Serum short-chain fatty acids (SCFAs) levels in middle-aged group. The group of 16 months old (16 M) was not treated with probiotics, which was considered middle-aged mice. We began the probiotic treatment from the 10th month of age and continued the treatment for 6 months. Resveratrol was used as positive control for antiaging intervention. Serum short-chain fatty acids (SCFAs) were measured after sacrificing including acetate, propionate, isobutyrate, butyrate, valerate, caproate, hexanoic acid, octanoic acid, and decanoic acid. Treatment groups were compared with the 16 M group (vehicle control). *P < 0.05, **P < 0.01, ***P < 0.001

SCFA belongs to the nature of the metabolites produced by probiotics. Next, the serum SCFAs levels were measured among different treatment groups (Fig. 5). Comparing to non-treatment control (16 M mice), resveratrol, high-dose probiotic treatment and high-dose probiotic plus postbiotic in serum SCFAs. High-dose probiotic treatment significantly increased the serum butyrate (478.8%, *P* < 0.001), caproate (135.9%, *P* < 0.05), octanoic acid (201.5%, *P* < 0.01) and decanoic acid (280.1%, *P* < 0.01) levels. The high-dose probiotic plus postbiotic would significantly elevate serum butyrate (518.5%, *P* < 0.001), isovalerate (168.9%, *P* < 0.05) and caproate (126.3%, *P* < 0.01).

## Discussion

In this study, we used a natural aging method to establish a 16-month-old murine model. Numerous studies have administrated D-galactose to establish an aging mice model, which successfully accelerated aging in 45 days [20–22].

Navarro et al. indicated that SOD levels and behavioral activity are significantly decreased in aging mice, whereas levels of the oxidative marker MDA are significantly increased in the mouse brain [23], which consistent with our natural aging model. Furthermore, levels of oxidative stress markers including MDA), protein carbonyl after protein oxidation, and mitochondrial 8OHdG decreased in mice receiving the probiotics (Fig. 2; Supplemental Figs, 2-5).

Mitochondria play an essential role in energy production through oxidative phosphorylation and intracellular homeostasis. Damage to or dysfunction of mitochondria can lead to aging, cardiovascular disease, neurodegenerative disorders, and cancer, among other outcomes [24]. Thus, the predominant forms of the free radical–induced oxidative marker mitochondrial DNA 8OHdG have been widely used as a biomarker of oxidative stress [25]. Our results indicate that mixed probiotic strains significantly reduced mitochondria 8OHdG levels in the brain, liver, and kidney (Supplemental Figs, 2-5). However, there was no large difference between the number of probiotics in the low-dose group (1.03 × 10^9^ CFU/kg) and the high-dose group (4.1 × 10^9^ CFU/kg) in antioxidative activities. More probiotic dosages should be test in the antioxidative assays in the future.

Postbiotics are fermentation components generated by bioactive probiotic strains. They consist of various metabolites including microbial cell fractions, short-chain fatty acids, teichoic acid, extracellular polysaccharides, peptidoglycan-derived muropeptides, and functional proteins, and they reportedly benefit the regulation of anti-inflammatory and immune effects [26]. The synergetic effects of *L. plantarum* and β-glucans were reported to enhance digestive enzyme activity and intestinal morphology [27]. Our results also confirmed the synergetic effects of viable probiotic strains combined with their postbiotics in elevating antioxidative activities in the brain, liver, heart, and kidney of middle-aged mice (Fig. 2; Supplemental Figs. 2-5).

Gut microbiota have been recognized as playing a key role in aging and in antiaging interventions [28]. Evidence of the potential beneficial effects of dietary probiotics in older adults continues to accumulate [29]. In our study, compared with the young group, the proportions of the *Bacteroidates* phylum, *Verrucomicrobia*, and *Actinobacteria* were smaller in middle-aged mice, whereas the amount of *Firmicutes* was elevated (Fig. 3a) The genera of *Lactobacillus, Bifidobacterium*, and *Akkermansia* exhibited a similar tendency after treatment with probiotic formulas (Fig. 3b). The probiotic-fed groups had increased gut *B. animalis*, *B. breve*, *B. bifidum*, and *L. plantarum,* especially the high-dose probiotics plus postbiotics group, which implies the fed probiotic strains successfully colonized the GI tract and enlarged the populations of the original species (Fig. 4a, b). Unexpectedly, the populations of some species not members of the fed probiotics also increased in the gut, including *L. johnsonii,* and *Akkermansia muciniphila* (Fig. 4a; Supplemental Fig. 6)*.*

The abundance of *L. johnsonii* notably increased to 4.42% in the gut of mice receiving probiotic (high-dose) plus postbiotics (Fig. 4c). Several beneficial functions of *L. johnsonii* have been reported, including thickened mucous membranes in stomach ulcers [30], restored numbers of serum IgA, IgG, and CD8 + cells, increased splenocyte counts in aged mice with protein–energy malnutrition [31], decreased glucagon and glucose levels in diabetic rats [32], and prevention of memory dysfunction [33]. The proportion of gut *Akkermansia* was also significantly elevated, to 0.41%, in mice receiving probiotics (high-dose) plus postbiotics, whereas in the 16-month-old mice it was only 0.04% (Fig. 4b). The colonization of *Akkermansia* in the gut was reported to relieve appendicitis-related inflammation and inflammatory bowel disease [34]. A recent clinical study discovered that *Akkermansia* were significantly increased among semisupercentenarians (age 105–109 years), which suggests that *Akkermansia* might play a part in building new gut homeostasis in extreme aging people [35].

A dysfunction in the gut–brain axis has been explained by a series of studies linked to neuropsychological, metabolic, and gastrointestinal disorders. Study findings reveal that perturbations of short-chain fatty acid and amino acid metabolism in serum and CSF are implicated in the onset of depression [36]. At the present study, the high-dose probiotics and high-dose probiotics plus postbiotics would significantly elevate serum butyrate levels (Fig. 5). Butyrate, a four-carbon short-chain fatty acid, is a crucial energy source for gut [37]. Studies had revealed multiple benefits of butyrate in human including enhancement of intestinal barrier function and mucosal immunity [38], elevating anti-inflammation status [39], modulating oxidative stress in the colonic mucosa [40] and alleviation of depression-related symptoms [41]. Thus, the probiotic secreting SCFAs may be the key factors in antiaging effect.

In conclusion, the mixed probiotic formula of *B. animalis* subsp. *infantis* BLI-02, *B. breve Bv889*, *B. bifidum* VDD088VDD088, *B. animalis* subsp. *lactis* CP-9, and *L. plantarum* PL-02 successfully elevated antioxidative activity with positive modulation of beneficial intestinal microbiota and elevated serum SCFA in the middle-aged mice. However, the sample sizes of each experimental group should be enlarged to eight mice, and animal behavior testing should be performed in the future. Finally, a human study on antiaging function could be conducted using the mixed probiotic formula of this study [42].

## Supplementary Information

Below is the link to the electronic supplementary material.

Supplementary file1 (DOCX 446 kb)

## Data Availability

Not applicable.
