# Supplementary material for: Probiotics and their Metabolites Reduce Oxidative Stress in Middle-Aged Mice
Source: Curr Microbiol. 2022 Feb 14;79(4):104. doi: 10.1007/s00284-022-02783-y (PMC8843923; doi:10.1007/s00284-022-02783-y)
Supplement: Supplementary file 1 — Supplementary file1 (DOCX 446 kb) [file 284_2022_2783_MOESM1_ESM.docx]

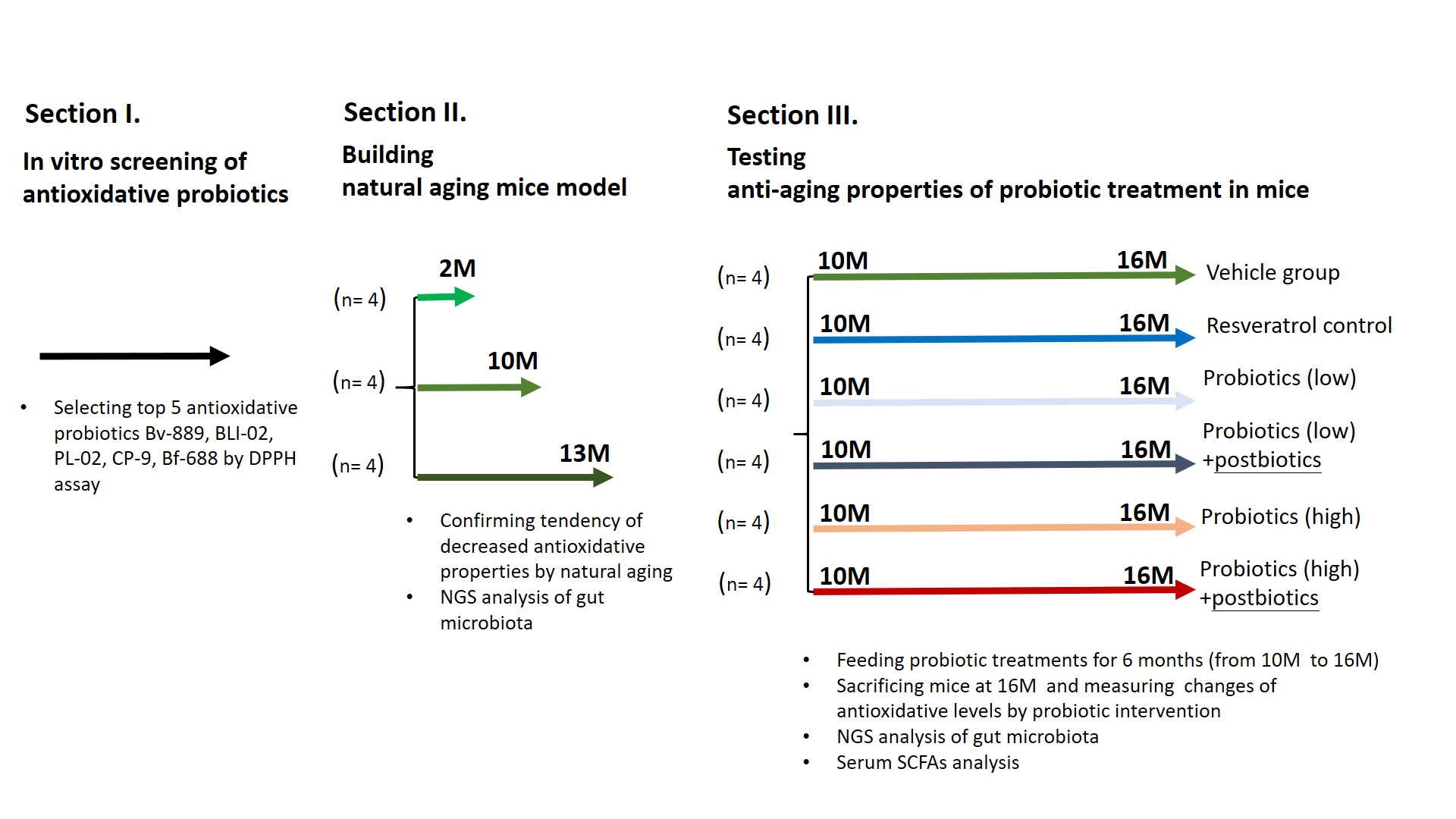


**Supplemental Figure 1. Experimental flowchart**

**Supplemental Figure 2. Probiotics elevated antioxidative activity in the brain (probiotics administered for 6 months)** (a) superoxide dismutase (SOD) assay (b) glutathione peroxidase (GPx) assay (c) catalase (CAS) assay (d) protein carbonyl assay (e) 8-OHdG assay. Experimental mice were divided into nine groups. The groups 2 months old (2M), 10 months old (10M), 13 months old (13M), and 16 months old (16M) were not treated with probiotics. The 2M mice were considered young, and those aged 10 months or more were considered aged. We began probiotic treatment with mice aged 10 months, and the treatment continued 6 months. Treatment groups were compared to the 16M group (vehicle controls). **p* < 0.05, ***p* < 0.01, ****p* < 0.001

**Supplemental Figure 3. Probiotics elevated antioxidative activity in the heart (probiotics administered for 6 months)** (a) superoxide dismutase (SOD) assay (b) glutathione peroxidase (GPx) assay (c) catalase (CAS) assay (d) protein carbonyl assay (e) 8-OHdG assay. Experimental mice were divided into nine groups. The groups 2 months old (2M), 10 months old (10M), 13 months old (13M), and 16 months old (16M) were not treated with probiotics. The 2M mice were considered young, and those aged 10 months or more were considered aged. We began probiotic treatment with mice aged 10 months, and the treatment continued 6 months. Treatment groups were compared to the 16M group (vehicle controls). **p* < 0.05, ***p* < 0.01, ****p* < 0.001

**Supplemental Figure 4. Probiotics elevated antioxidative activity in the liver (probiotics administered for 6 months)** (a) superoxide dismutase (SOD) assay (b) glutathione peroxidase (GPx) assay (c) catalase (CAS) assay (d) protein carbonyl assay (e) 8-OHdG assay. Experimental mice were divided into nine groups. The groups 2 months old (2M), 10 months old (10M), 13 months old (13M), and 16 months old (16M) were not treated with probiotics. The 2M mice were considered young, and those aged 10 months or more were considered aged. We began probiotic treatment with mice aged 10 months, and the treatment continued 6 months. Treatment groups were compared to the 16M group (vehicle controls). **p* < 0.05, ***p* < 0.01, ****p* < 0.001

**Supplemental Figure 5. Probiotics elevated antioxidative activity in the kidney (probiotics administered for 6 months)** (a) superoxide dismutase (SOD) assay (b) glutathione peroxidase (GPx) assay (c) catalase (CAS) assay (d) protein carbonyl assay (e) 8-OHdG assay. Experimental mice were divided into nine groups. The groups 2 months old (2M), 10 months old (10M), 13 months old (13M), and 16 months old (16M) were not treated with probiotics. The 2M mice were considered young, and those aged 10 months or more were considered aged. We began probiotic treatment with mice aged 10 months, and the treatment continued 6 months. Treatment groups were compared to the 16M group (vehicle controls). **p* < 0.05, ***p* < 0.01, ****p* < 0.001

**Supplemental Figure 6. NGS analysis of *Akkermansia muciniphila* change by taking probiotic product.** The 2M group was considered young. Experimental mice were divided into nine groups. We began probiotic treatment with mice aged 10 months, and the treatment continued 6 months. Treatment groups were compared to the 16M group (vehicle controls). **p* < 0.05, ***p* < 0.01, ****p* < 0.001
